# Literature Review of Deep-Learning-Based Detection of Violence in Video

**DOI:** 10.3390/s24124016

**Published:** 2024-06-20

**Authors:** Pablo Negre, Ricardo S. Alonso, Alfonso González-Briones, Javier Prieto, Sara Rodríguez-González

**Affiliations:** 1 BISITE Research Group, Universidad de Salamanca, Patio de Escuelas, 37008 Salamanca, Spain; alfonsogb@usal.es (A.G.-B.); srg@usal.es (S.R.-G.); 2 AIR Institute, Av. Santiago Madrigal, 37008 Salamanca, Spain; ralonso@air-institute.com; 3 UNIR (International University of La Rioja), Av. de la Paz, 137, 26006 Logroño, Spain

**Keywords:** video violence detection, artificial intelligence, surveillance camera, action recognition, computer vision

## Abstract

Physical aggression is a serious and widespread problem in society, affecting people worldwide. It impacts nearly every aspect of life. While some studies explore the root causes of violent behavior, others focus on urban planning in high-crime areas. Real-time violence detection, powered by artificial intelligence, offers a direct and efficient solution, reducing the need for extensive human supervision and saving lives. This paper is a continuation of a systematic mapping study and its objective is to provide a comprehensive and up-to-date review of AI-based video violence detection, specifically in physical assaults. Regarding violence detection, the following have been grouped and categorized from the review of the selected papers: 21 challenges that remain to be solved, 28 datasets that have been created in recent years, 21 keyframe extraction methods, 16 types of algorithm inputs, as well as a wide variety of algorithm combinations and their corresponding accuracy results. Given the lack of recent reviews dealing with the detection of violence in video, this study is considered necessary and relevant.

## 1. Introduction

Physical aggression affects almost every aspect of life, not only the direct victims but also their families, society in general, and day-to-day life. It also affects the economy (shopping, travel, tourism, etc.) [1] and the mental health of citizens because of the permanent state of insecurity [2]. Some studies point to alarming data, for example, physical assault is the major cause of mortality among 15–44-year olds around the world [3]. The research carried out by Hillis et al. (2016) [4], which has been published in the *American Academy of Pediatrics*, states that at least 50% or more of children in Asia, Africa, and North America experienced violence in 2015, and that globally, more than a half of all children (1 billion children, aged 2–17) experienced such violence. According to a study carried out by the European Union Agency for Fundamental Rights (FRA), more than one in four Europeans was a victim of harassment and 22 million were physically assaulted in one year [5]. For these reasons, it is necessary to address the issue of physical aggression in societies around the world.

Multiple long-term, medium-term, and short-term solutions have been proposed. Those for the long term consist of understanding reasons or situations that exacerbate violence, so that future generations can be educated to avoid them. Some studies have studied an increase in violence due to exposure to an environment of violence and aggressiveness, which leads to a tendency to use violence as a tool [6]. It has also been shown that there is a direct correlation between aggression and a poor maternal relationship or low self-esteem [2]. Moreover, technologies, such as smartphones, contribute to an increase in violence, as does the consumption of aggressive video games [7].

It has also been observed that rapid urbanization, coupled with population growth, has led to more crime [1]. Many studies also talk about how COVID-19, more specifically the consequences it has had on people’s lives, has led to an increase in violence, producing a “perfect storm” of factors [8]. According to Killgore et al. (2021) [8], domestic violence cases increased by 7.5% in the first five weeks following the imposition of social distancing measures [9]. In addition, there was a rise in reports of male violence in the first few months of the pandemic [10,11].

Other studies have opted for medium-term solutions to face violence by trying to relate street population density and the urban landscape to crime rates. Mobile phone and social network data have been used to measure population density in urban areas and to relate it to the crime rate using statistical techniques focused on spatial analysis [12], although the fact that it is not possible to differentiate between indoor and outdoor populations is a drawback. Moreover, through the use of convolutional neural networks (CNNs), it has been discovered that crime rates are lower in green areas [13]. Another study employed deep learning to assess images from Baidu Street View (the namesake of Google Street View in China) showing the number of people on the streets, as well as the type of urban landscape (buildings, green areas, etc.) and correlated them with the number of crimes [14]. Another study also used Google Street View images to analyze the number of vehicles and the pavement, and related those features to the crime rate through the use of machine learning techniques [15].

Those medium-term methods that analyze and compare the amount of people or traffic on the streets and the type of urban landscape with the crime rate provide real and specific information on which areas are more prone to events of physical assault and how to build new urban landscapes that are less crime-inducing; however, it is an offline approach that does not help victims of assault in real time. The short-term solutions and the last-minute prevention of physical assault are precisely the ones which operate in real time; and they are the subject of this review.

So far, the use of images and security cameras has largely been used to provide post-crime evidence, which is used as proof to detect the culprits and is useful for insurance companies, police, or legal proceedings [16]. However, there are already several published papers that have developed real-time violence detection solutions. These solutions are based on the use of security video cameras driven by artificial intelligence (AI) algorithms [17,18,19]. This is a growing field [20] whose development has been possible due to the evolution of three main pillars: the increased use of images and security cameras [21,22], the technological development of big data platforms [23,24], and the development of artificial intelligence algorithms [25] that allow for the analysis of images and video [26].

The installation of security cameras has increased around the world for monitoring and security reasons, among others [21,22]. It is important to highlight that while the use of images and video has a huge number of applications that can improve the quality of life of citizens, there are already studies which address the risk to personal privacy posed by large-scale recording [27]. Big data platforms currently offer us the ability to obtain and process a large amount of data from the environment that surrounds us [23,28]. These tools allow us to capture, store, and analyze, in real time, different types of information, such as images and videos [24]. Finally, artificial-intelligence-based algorithms for image and video analysis have become more commonplace in recent years; they have also grown in variety and accuracy [26].

It is worth asking ourselves whether the use of artificial intelligence is necessary for the detection of aggression. The reality is that the constant analysis of images and video by humans involves either a high cost of personnel (salaries, facilities, etc.) or a low level of supervision, in the case of a few people monitoring a large number of scenarios, which can lead to the loss of lives [29]. This is why the use of AI image and video analysis techniques plays a crucial role in the development of effective violence detection solutions.

The detection of violence in videos falls within the field of computer vision, specifically in the area of action recognition [30]. Computer vision is a branch of artificial intelligence that enables computers to interpret and make decisions based on visual data. Action recognition is a subfield that focuses on identifying specific actions within video sequences [31]. Using AI for violence detection in videos involves training models to recognize patterns and behaviors indicative of violent activity [3]. AI-based violence detection in videos works by training algorithms on labeled video datasets, where violent and non-violent actions are annotated. These algorithms learn to recognize patterns and features associated with violent behavior, enabling them to identify such actions in new, unseen videos [32].

In conclusion, physical aggression represents a societal concern with broad implications. Numerous research endeavors have been undertaken to address this issue through various approaches, each providing solutions with varying degrees of directness. The real-time identification of violence stands out as the most immediate and critical solution, serving as the ultimate line of defense in identifying individuals subjected to physical harm. This achievement owes its feasibility to several key factors: the escalating utilization of security images and videos, the advancement of large-scale data platforms, and the evolution of algorithms capable of image and video analysis empowered by artificial intelligence.

The aim of this work is to extend a systematic mapping study [32], with the aim of providing a comprehensive and current view of AI-powered video violence detection, focusing particularly on physical assault. The valuable contribution of this paper lies in the development of a comprehensive and up-to-date review, covering all the steps involved in video violence detection using artificial intelligence. Another unique contribution of this paper is the analysis of the current characteristics of AI-based violence detection in video. This paper categorizes and ranks these characteristics on the basis of the number of articles in which they are mentioned. Lastly, an element that distinguishes this paper is its analysis of the types of algorithms used in video violence detection, the combinations of algorithms employed, and the results obtained for the most commonly used datasets in the state-of-the-art literature.

This work is structured as follows: Section 2 discusses the reviews published in the last two years that address the topic of AI-based violence detection in video. Section 3 summarizes the 63 selected articles on AI-based violence detection published in the last three years, grouped according to the type of algorithm used for violence detection. Section 4 presents the grouping and categorization of all violence detection phases using the selected articles. Finally, Section 5 draws conclusions from the findings and describes future work.

## 2. Related Work

The contents of past reviews are discussed in this section, highlighting their strong and weak points. This is to help us carry out a review that is comprehensive and contributes to the current state of the art.

Yao and Hu [33] highlighted the challenge of defining violence due to its inherent ambiguity and infrequent occurrence, making real-case recordings hard to obtain. They discussed the disparity between conventional methods and deep learning (DL) techniques for detecting aggression but did not provide details about the article selection process, including databases, search criteria, time frame, and filters, nor did they reference related works. The review categorized state-of-the-art articles into two sections: the traditional framework and deep learning. The traditional framework section was further divided into feature extraction techniques and classifiers. It compiled articles using key feature extraction methods (motion, appearance, trajectory-based, and feature descriptors) and described works using support vector machine (SVM) and k-nearest neighbors (KNN) classifiers, outlining their strengths and weaknesses. The paper provided a table in which the articles were listed, specifying the scene type (crowded or not) and feature extraction techniques, but not the classifier or video class used in each study.

Siddique et al. [31] provided an overview of violence detection, outlining basic steps and concepts with a visual scheme and summary table. While not a systematic review, the paper included a comprehensive research methodology, detailing search queries, databases, date ranges, and filtering processes. It categorized aggression detection algorithms into 19 sections, though some sections had only one citation, suggesting a need for broader grouping. The summary table detailed object detection, feature extraction, scene congestion, and accuracy; however, it lacked information on classifier types and datasets, and did not compare algorithm performance. Additionally, the feature extraction table lacked grouping by feature type, and the dataset table provided minimal information beyond clip counts and publication years.

Kaur and Singh [34] summarized recent reviews on aggression detection since 2016 in a conference paper. They categorized the reviewed papers into two main groups: traditional methods, further subdivided by feature extraction and classification techniques; and deep learning methods. The paper highlighted studies using convolutional neural networks (CNNs) and long short-term memory (LSTM) as recent and promising techniques in the field. Various challenges in aggression detection were discussed, including issues such as changes in illumination, overlapping objects, and confusion with other actions. The paper concluded that there was no universal solution to these challenges collectively and suggested that, instead, they should be addressed on a case-by-case basis.

Shubber et al. [3] lacked a related work section and a dedicated research methodology. The reviewed literature was categorized into traditional methods and deep learning methods, with deep learning showing higher precision. The paper detailed feature extraction and classifiers, providing tables for feature extraction methods across different studies and four classification methods with corresponding citations. In the deep learning section, the paper highlighted that advancements were enabled by abundant data availability and high processing power. It also included a section listing datasets used in the reviewed works, detailing video clip counts, frame numbers, and content types (e.g., hockey videos, films, real scenes). However, it did not specify whether these datasets were crowded. Tables for each dataset showed the accuracy percentages achieved across numerous studies.

Omarov et al. [20] provided a comprehensive systematic review of violence detection, organized into five main sections covering research methodology, fundamental concepts, classification of detection techniques, video features and descriptors, datasets, evaluation parameters, and challenges in violence detection from 2015 to 2021. The research methodology section outlined systematic review elements such as research questions, article definition, and filtering steps. Basic terms were defined, and the process of training and testing physical aggression detection algorithms was explained. An innovative aspect of the paper was a section on the parameters used in assessing physical aggression detection. Associating these parameters with specific studies could enhance understanding.

On the whole, the analyzed reviews, which were published in the last three years, do not cover all the aspects of violence detection. This points to the need for an updated review that provides a comprehensive insight into AI-based violence detection in video.

## 3. Selected Article Description

**Figure 1:**
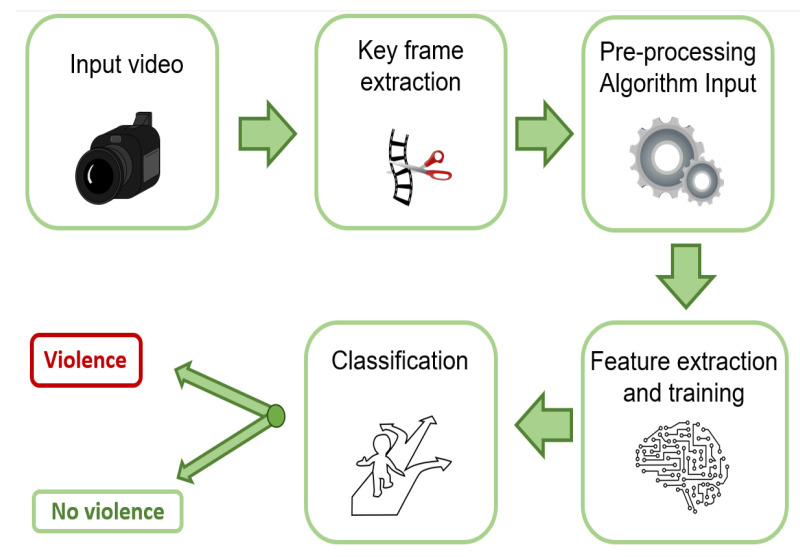
Basic steps of video violence detection algorithms.

This section provides a brief explanation of the articles selected for the systematic mapping study [32], and how they have been organized and analyzed in this paper. The main focus has been on the selected papers’ use of algorithms for feature extraction and training. The selected articles are grouped into seven subsections, according to the type of algorithm used to detect violence. Before presenting the selected articles, it is important to explain the basic steps of an algorithm for violence detection in video. A graphic scheme has been made to help illustrate these steps in Figure 1.

The first step starts with the input of a video. To this end, a dataset of videos which contain violent and non-violent scenes is necessary. The second step is keyframe extraction, although not all architectures include this step. It consists of the selection of frames that may potentially contain violence; this selection is performed in order not to have to process large amounts of video, thereby reducing the required computation levels. The third step consists in transforming the data to serve as input to the violence detection algorithm, depending on the features the algorithm is to extract. The fourth step is feature extraction and training the algorithm on these features; as discussed further on in Section 4.6, there are different combinations of algorithms, and therefore, there are variations in the process. Finally, a classifier decides whether the scene is violent or non-violent.

The models presented in the selected articles have been organized into categories according to the algorithms used for the violence detection. It has been decided to group them in the following seven categories. There are articles that combine two types of algorithms, and they have been classified here as *phase 1* and *phase 2*. In this paper, the categorization of the different violence detection algorithms is mainly based on the type of algorithm used (manual feature, CNN, LSTM, transformers). However, additional categories have been created for skeleton-based and audio-based approaches; even though they may use deep learning methods or manual methods, their approach is clearly different, focusing on detecting humans in video or using another data dimension such as audio. Additionally, another category has been created for those articles that combined CNN and LSTM; this is due to the large number of works that used this combination [32].

- **CNN + LSTM**: Articles that use a combination of convolutional neural networks (CNNs), independently of whether they are pre-trained or not, and long short-term memory (LSTM). CNNs extract spatial features, and these features are fed as input to the LSTM to extract temporal features. There are two distinct phases, with the LSTM being fed from the features extracted by the CNN and the LSTM [35,36].
- **CNN**: Articles that rely exclusively on CNNs for violence detection in videos. CNNs extract spatial features from video frames and they are used to make classification decisions (although, as shown further on in the paper, in some of the selected articles, the use of CNN-3D and CNN-4D did not allow for the extraction of temporal and spatial patterns [37]). The use of pre-trained CNNs for violence detection has also become very popular; those have been previously trained on big image datasets, such as ImageNet, to initialize their initial weights. These models are then fine-tuned to extract relevant features and detect violence patterns in video sequences, leveraging transfer learning to enhance efficiency and accuracy [38].
- **Manual feature**: Includes the articles in which either only feature extraction or both feature extraction and training have been performed with manual feature extraction techniques (those whose nature is mathematical but not based on machine learning or deep learning techniques) [39,40].
- **Skeleton-based (deep learning or manual)**: Includes works in which the algorithms focus on the skeleton-based concept, which consists of detecting the body position of the people appearing in the video, and from these positions, extrapolate whether or not there is violence. This group includes articles in which this process is performed with both mathematical or deep learning algorithms, since unlike those mentioned above, they do not focus on changes in movement, textures, or lighting, as in manual algorithms, or on the extraction of spatial features, as in CNNs, but their focus is the detection of body positions [41,42].
- **Audio-based (DL or manual)**: Articles that use video audio as a tool for violence detection. The included works may not necessarily rely only on audio as the input for algorithms, but it is a noteworthy element due to its distinct focus [43,44].
- **Transformer**: Articles that apply transformer-based architectures for detecting violence in videos [45,46].
- **LSTM**: Articles that use LSTMs to extract temporal patterns of violence in video [47].

### 3.1. CNN + LSTM

Talha et al. [35] developed a very fast and efficient real-time violence detection system, which was tested on the personal devices of the system developers. It is based on a CNN which extracts spatial features. These features feed an LSTM. The CNN also uses two fully connected layers as a classifier. Madhavan [36] developed a model, without presenting its performance results. The model was intended to address the challenges associated with classification in inconsistent weather and illumination conditions. This approach also potentially solved the problem of dedicating a low amount of pixels for video classification. Ullah et al. [48] used Mask R-CNN for feature frame selection, which is an extension of the Faster R-CNN model based on object detection (in this article it was used to detect people and cars). It has the added ability of detecting and labeling segment objects in an image. For feature extraction, the authors used two CNNs: DarkNet and a CNN which receives as residual input optical flow. With these extracted features, a multilayer long short-term memory (M-LSTM) was fed. Vijeikis et al. [49] generated a model based on a CNN and LSTM, which made it computationally light and fast; nevertheless, it had slightly lower results in terms of accuracy. Halder and Chatterjee [50] used a lightweight convolutional neural-network-based bidirectional LSTM to identify violent activities with excellent results.

Traoré and Akhloufi [51] used a pre-trained VGG-16 with INRA person dataset to extract spatial features, which then feeds a type of RNN called BiGRU (bidirectional gated recurrent unit). Finally, as a classifier the authors used three fully connected layers, the last one with softmax activation. Similarly, Ref. [52] used VGG-16 for spatial feature extraction, and an LSTM was used for temporal feature extraction. Asad et al. [53] proposed a violence detection model that took as inputs two video frames at times *t* and *t* + 1 (not the subtraction of both). Two pre-trained CNNs were used, one receiving frame t and the other *t* + 1. They were used to extract high- and low-level features. Additional wide dense residual blocks (WDRBs) were used to learn these combined feature maps from the two frames, and then, the LSTM network learned the temporal patterns between the features extracted by the CNNs. To raise an alarm that violence detection is occurring, a real-time graph was plotted with the level of violence obtained from the algorithm output and, above a certain value, the scene was identified as violent. Contardo et al. [54] used a CNN pre-trained with ImageNet called MobileNetV2, which extracted spatial features and whose output was fed into two LSTMs to test which one obtained better results: a temporal Bi-LSTM and a temporal ConvLSTM.

Gupta and Ali [55] used the VGG-16 pre-trained network whose output was fed into an LSTM and a Bi-LSTM, in order to test which of the two yielded better results. Islam et al. [56] added a multitude of parameters describing the datasets used (total classes, number of videos per class, frames per second, video length, avg. frames per video, resolution, number of locations). It focused on sexual assault detection, but also on physical assault detection. The authors used pre-trained VGG-16 and VGG-19, whose output they fed into an LSTM. Jahlan and Elrefaei [57] selected frames randomly from each frame packet, furthermore they converted the images to squares by choosing any area of the dataframe in between. As input of the CNN, the selected frames were not inserted, but the difference between frame *i* and *i* + 1 was inserted. The CNN that was used was automated mobile neural architecture search (MNAS) which is a lightweight CNN. A convolutional LSTM was used not only on temporal extraction; instead, the convolutional layer added a spatial element. Three different classifiers were used to see their effectiveness; prior to the classifier, the results of the trained features were scaled between 0 and 1 (normalized). The best performing classifier was the SVM.

Mumtaz et al. [58] used VGG-19 (pre-trained CNN) whose output they fed into an LSTM. At the time of publication of the paper, and to the best of the authors’ knowledge, they were the first to introduce a process control technique: control charts for surveillance video data analysis. The concept of control charts was merged with a novel deep-learning-based violence detection framework. Sharma et al. [59] used a CNN pre-trained with ImageNet for spatial feature extraction called Xception, the output of which is fed into an LSTM. Singh et al. [60] separated the state of the art between motion-based, machine learning, and deep learning. They used two CNNs to extract low-level features and local motion features. The result was passed to an LSTM that learned global temporal features. It was mentioned how the extracted features included edges or lines of objects and people, and body motions. These features also included appearance-invariant features such as changes in illumination, weather, and other environment or background related changes.

Srivastava [61] proposed two different algorithms. One of them was a violence detection algorithm. The second one was a facial identification algorithm, useful in case violence occurs. It focused on violence detection using drone cameras. Spatial features were extracted with a CNN block using architectures that had been pre-trained with ImageNet and whose output was fed into an LSTM. In total, seven different algorithms were used in addition to three combinations of some of them. Traoré and Akhloufi fed two CNNs called EfficientNet, which were pre-trained on ImageNet, one of them with optical flow and one of them with RGB. The outputs of these two CNNs were fed into an LSTM, and finally, to a classifier, consisting of an FCL with a sigmoid activation layer.

Islam et al. [62] proposed a two-input structure that combined a CNN with a separable convolutional LSTM (SepConvLSTM). One of the inputs receives as input the RGB video with background suppression and the other one the frame difference (difference between frame *i* and *i* + 1). They also implemented three versions of the proposed architecture in which the classification functions vary, as well as the information that is passed to the fully connected layers. Mugunga et al. [29] decided to introduce optical flow images which, although they did not contain all the scene information, helped to reduce the computational cost and to extract spatio-temporal features. This output fed a Bi-ConvLSTM that was able to extract short- and long-term features, obtaining better results than unidirectional ConvLSTMs.

### 3.2. CNN

Mahmoodi et al. [63] presented a work where an image segmentation method called SSMI was used to avoid introducing all the frames of the video to the CNN. Then, with a single 3D-CNN structure it performed spatio-temporal-focused feature extraction and used fully connected layers as the classifier. Ahmed et al. [64] used a CNN-v4, highlighting that, while other state-of-the-art work used CNNs for violence detection, it introduced all the frames of the video, which was very expensive computationally, and therefore, the selection of characteristic frames for much lighter computation was essential. Ji et al. [65] presented a new dataset called *Human Violence Dataset* containing 1930 video clips from movie promotion videos on YouTube. However, it did not consider only physical aggression, but also gun violence. The two-stream CNN model is a neural network architecture that uses two independent streams of visual information: one for spatial information and one for temporal information. The temporal stream receives a single image, while the spatial stream receives 10 frames of optical flow. After extracting features with a CNN, a machine learning algorithm is trained to quantify the violence in the videos by optimizing the weights. The article quantified levels of violence in the processed videos through feature calculation, which it based on a ranking score. Three levels of violence (L1, L2, and L3) were determined using a confusion matrix.

Ehsan et al. [66] highlighted the advantages of CNNs, where feature extraction and feature training was performed within the CNN architecture itself and not in separate algorithms. The proposed CNN architecture was called Vi-Net, which performed classification in two fully connected layers, the latter of these with the softmax activation function. Jayasimhan et al. [67] proposed a 3D-CNN followed by a 2D-CNN. It did not require transfer learning (pre-trained network) or manual feature extraction, which made the structure lightweight. The 3D layer could capture temporal information over time. The 2D layer aimed to fuse temporal features into a 2D representation. Kim et al. [68] proposed several methods to improve people tracking, as current methods still present difficulties. A 3D-CNN structure was proposed for fall detection and violence detection.

Monteiro and Durães [69] used the AVA dataset, selecting 17 violent actions out of 80. Their model had two paths: a slow one for appearance details and a fast one for dynamic motion. Each path used a ResNet. Features from both paths were combined using global average pooling and a fully connected layer, followed by softmax. Then, fine-tuning was performed with the X3D network to refine the results, also resulting in a fully connected layer. Talha et al. [70] divided the related work into deep learning, supervised and unsupervised learning, knowledge distillation, and multimodal learning models. The authors proposed a model using a C3D with a classifier from a fully connected layer, with the sigmoid activation function for the last layer.

Appavu [71] used a keyframe selection method for each video sequence: one frame for spatial, three for temporal and six for spatio-temporal. Multiple inputs were introduced to the CNN for the spatial, temporal, and spatio-temporal branches: grayscale equation, optical flow constraint equation, and differential kinetic energy image (adaptive mean thresholding [AMT], adaptative Gaussian thresholding [AGT]). It is important to highlight the use of gradcam (widely used XAI technique) to illustrate the input of the spatio-temporal branch of the CNN, although the algorithm only used it to facilitate the reader’s understanding and not for a real purpose of explainable artificial intelligence.

Adithya et al. [72] used a pre-trained CNN for feature extraction to improve the final weights. It obtained good results. Several combinations of CNN (5, 8, and 10 convolutional layers) were made, where the 8-layer combination gave good results. Finally, the best combination was the five-layer 3D-CNN network. Bi et al. [73] used a selected number of relevant frames to filter out false positives (hugs or other actions that may look like violence). For feature extraction, ResNet18 was used (pre-trained CNN), which is a type of CNN that is easier to train and has fewer parameters than CNN-3D and LSTM. To facilitate feature extraction, an image segmentation method (Deeplab-V3plus) was used. It was argued that reducing the dimensionality of the images extracted from the datasets would allow a much more clustered feature space. Image segmentation could focus on the explainability of the algorithm, although this approach was not discussed in the paper.

Chen et al. [38] used the detection of changes in brightness as a method of extracting characteristic frames. This is because the difference in brightness between adjacent images could indicate a change in the action of the video. Where the maximum variation in brightness determined segmentation. As an algorithm for feature extraction and training, a combination of pre-trained networks between ResNet and Inception-V1 was used. Freire-Obregón et al. [74] used YOLOv4 (which detects and labels an object in an image) and SiamRPN (which focuses on tracking an object) for the extraction of characteristic frames. For violence detection, a 3D-ConvNet with two inputs was used, which was also pre-trained by the Kinetics dataset algorithm. In addition, different classifiers were evaluated to assess which one obtains the best accuracy with the proposed structure. The effect of the environment on the accuracy of the algorithm was analyzed, showing that footage without context produces a deterioration of the classifier between 2 and 5%. The authors also showed that performance stabilizes for context-free sequences, regardless of the level of context constraint applied. In addition, the article evaluated training and testing with different combinations of the datasets used. It was observed that better results were obtained when using the same dataset for training and testing.

Gkountakos et al. [75] used a pre-trained 3D-ResNet with fully connected layers for classification. Huszár et al. [76] proposed two pre-trained architectures: Fine-tuned X3D-M model and transfer-learned X3D-M model. The fine-tuned X3D-M model optimized the X3D-M parameters learned from the Kinetics-400 dataset, while the transfer-learned X3D-M model extracted spatio-temporal features first, without modifying the X3D-M parameters, to train multiple fully connected layers. Better results were obtained with the fine-tuned X3D-M model. Jain and Vishwakarma [77] used *dynamic image* as input to the algorithm, which focused on the motion of salient objects in the video, combining and averaging background pixels with motion patterns while retaining long-term kinetics. Features were extracted with ResnetV2 pre-trained with ImageNet. Training was performed in the final layers of this algorithm along with fine-tuning techniques (freezing certain values of the neurons to adjust the weights).

Liang et al. [78] used YOLOv5 for the extraction of characteristic frames. YOLOv5 is an object detection algorithm, in this case, it was used to detect people in video. DeepSort was then used to track the person. Subsequently, a SlowFast network with ResNet pre-trained as the CNN was used for feature extraction and training. One of the challenges to be solved in violence detection involves the difficulty in identifying the different positions and angles of the subject with respect to the surrounding space. The following steps were used to solve this challenge: the exact positions of the subjects were determined in each video frame, the movement of the subjects was tracked over time, the location where violent actions occurred was identify and marked, these coordinates were mapped onto a real geographic space, and the spatial and temporal information from the video was fused, to represent violent behaviors on a geographic map, allowing for an accurate understanding of their location in the real world. Mumtaz et al. [79] proposed a violence detection method that used an architecture called Deep Multi-Net (DMN), which combined pre-trained convolutional neural networks (CNNs) such as AlexNet and GoogleNet with ImageNet. Violence detection was performed on sequences of images or frames taken from field hockey and movie datasets. DMN not only excelled in terms of accuracy outcomes, but it also demonstrated superior efficiency with rapid learning abilities, surpassing both AlexNet and GoogleNet, with a remarkable 2.28-fold speed advantage, all while maintaining comparable competitive accuracies.

Que et al. [80] focused on the detection of violence in long-duration videos using pre-trained CNNs. The authors focused on achieving accuracy in determining when the episode of violence began and ended since it is an understudied area in the literature. During the second phase, the deconvolution technique was employed to precisely pinpoint the potential video segment down to the individual frame, thereby establishing the exact moment of violence occurrence. It was emphasized that the preprocessing could be much better, as well as the method of detecting the start and end of the violence in the video. Santos et al. [81] asserted that 3D-CNNs are more advanced than conventional CNNs, being able to extract temporal as well as spatial information. The authors used a CNN pre-trained with Kinetics-400 called X3D neural network. Sernani et al. [82] repeatedly mentioned the importance of robustness on violence detection and how the AIRTLab dataset was specifically designed to test the robustness of the algorithms. The paper focused on testing whether its algorithms exhibited these characteristics or not. Three different DL algorithm structures were presented. The pre-trained 3D-CNNs were trained with the Sports-1M dataset. The authors highlighted that transfer learning can improve efficiency, rather than algorithms trained from scratch, and cited other work to prove this.

Shang et al. [83] divided the related work into deep learning models, supervised and unsupervised learning, knowledge transfer (knowledge distillation), and multimodal learning. Firstly, the authors proposed to transfer information from large datasets to small violent datasets based on mutual distillation with a pre-trained self-supervised model for RGB vital features. Second, the multimodal attention fusion network (MAF-Net) was proposed to fuse the obtained RGB features with stream and audio features to recognize violent videos with multimodal information. Magdy et al. [37] separated the video into frame packets and an optical flow-based technique was applied to detect areas with motion. The paper compared CNN-3D versus CNN-4D architectures. It stated that CNN-3D was good at analyzing short time spans, but CNN-4D also allowed for understanding more complex spatio-temporal relationships. The CNN had been pre-trained using ImageNet.

Hua et al. [84] proposed incorporating a residual attention module into the stacked hourglass network to improve human pose estimation by addressing the reduction in initial image resolution. The new architecture enhanced the resolution and accuracy of image features. The experimental results demonstrated that this approach achieved more accurate and robust human pose estimation in images with complex backgrounds. Liu et al. [85] proposed a novel method for maintaining temporal consistency in human pose estimation from video using structured-space learning and halfway temporal evaluation methods. By employing a three-stage multi-feature deep convolution network, the method ensured accurate and stable human pose estimation with superior long-term consistency across video frames.

### 3.3. Manual Feature-Based Approach

Wintarti et al. [39] selected 20 frames from each video sequence randomly as feature frame selection. For feature extraction, two methods were used: principal component analysis (PCA) (dimensionality reduction) and discrete wavelet transform (DWT), which consists of a set of sub-bands of signal frequencies for video processing. A support vector machine (SVM) algorithm was trained from the extracted features and the SVM itself acted as a classifier. Mohtavipour et al. [86] divided the related work into deep learning, global handcrafted, and local handcrafted. The energy difference was used as input to the algorithm. A CNN was trained that had three inputs. For each sequence, one frame was selected for the spatial, three for the temporal, and six for the spatio-temporal. The spatial focused on appearances and was converted to gray video, the temporal used optical flow, and the spatiotemporal generated a differential motion energy image. Lohithashva [40] used local orientation pattern (LOOP) as a manual feature extraction technique. These features were trained by an SVM which also acted as a classifier.

Jaiswal [87] used local binary pattern (LBP) and fuzzy histogram of optical flow orientations as manual feature extraction techniques. As a feature training technique it used AdaBoost (adaptive boosting) which is a machine learning technique. Finally, as a classifier it used a technique based on decision trees called Ensemble RobustBoost aggregation. Hu et al. [88] proposed two architectures. Starting from a video clip, the authors divided the video into three 3-dimensional arrays: X-T plane, Y-T plane, and X-Y plane. TOP-ALCM was then applied, which is an image processing and data analysis technique used to capture co-occurrence patterns of features in different directions and angles in a matrix. TOP-ALCMs are a matrix representation of these co-occurrences. From the TOP-ALCM results, two architectures were proposed, either the use of an SVM from the obtained elements such as entropy, energy, etc.; or the use of a CNN for training and classification.

### 3.4. Skeleton-Based Approach

Zhou [41] divided related work between human pose estimation (skeleton keypoints) and action recognition (where DL and traditional methods were introduced). In the initial feature extraction, each video frame was passed through a 3D convolutional neural network (3D-CNN), considering both spatial and temporal information across frames to capture action features. Then, features extracted by the 3D-CNN, such as HRNetW32, were divided into smaller patches to process manageable portions and capture fine details. Afterwards, each extracted feature patch was flattened into a one-dimensional vector, and position embeddings were added to account for positional relationships. Then, the TokenPose Model was used, where the sequence of patch vectors, along with special tokens representing keypoints in video actions, was fed into a transformer layer, capturing relationships between patches and keypoints. Finally, a multilayer perceptron (MLP) in the model’s head predicted keypoint heatmaps, using tokens from the final transformer layer as input, ultimately locating relevant keypoints in the video, followed by classification based on spatio-temporal features to determine if the video contained violent content. It is worth mentioning that saliency maps were applied to show how the algorithm selected the keypoints (articulations) of the people appearing in the video. This served as an explanation for the reader.

Hung et al. [42] divided the related work into deep learning models, supervised and unsupervised learning, knowledge distillation, and multimodal learning. They used a deep-learning-based method to obtain, from the introduced video, the number of skeletons, the distance between skeletons, and the changes in human skeleton hand acceleration. From these extracted features, an SVM was trained for the classification of violent and non-violent scenes. Naik and Gopalakrishna [89] made a different division of the related work: optical-flow-based methods, histogram of optical flow, space–time interest-point-based methods, and convolutional neural network methods. DeepPose was used for the extraction and training of the body positions of people appearing in the video, whose output fed an LSTM that extracted temporal relations.

Narynov et al. [90] classified the types of skeleton-based algorithms on RGB images into two categories: top-down algorithms and bottom-up algorithms. They used a pre-trained CNN called PoseNet to extract and train the body position of people appearing in the video. From the detected objects (people), skeleton features were extracted, and a temporal tracking of the extracted skeletons was carried out. The detection involved a distinction between punching and kicking. Srivastava et al. [91] proposed violence detection in a new dataframe created with images taken by a drone at a certain height from the ground. The paper involved identifying the human figure, selecting keypoints in their posture by using a CNN with two inputs. From those extracted and trained features, an SVM classifier was used which divided the violence detection into six features. Su et al. [92] extracted skeleton-based features, representing a geometric X, Y, Z map, where Z represented the temporal dimension. It was able to differentiate “heads” so that it could track the movements being made. These geometrical maps positions were taken to train SPIL (skeleton point interaction learning) for classification.

### 3.5. Audio-Based Approach

Mahalle and Rojatkar [43] established that the main objective for using audio feature extraction was to reduce the data dimensionality by extracting the most important features from audio samples. When the feature vector dimensionality is small, a set of features can be useful for representing the characteristics of audio samples. From the audio feature extraction, labels were assigned to each instance. All the labels with their audio were introduced to an extreme learning machine which was trained to identify violent audios. Wu et al. [93] presented a large-scale multi-scene dataset called *XD-Violence*. The model for violence detection involved a neural network comprising three concurrent branches aimed at discerning various connections among video fragments and merging attributes. The comprehensive branch grasped extensive dependencies through similarity precedence, while the localized branch seized local positional correlations using proximity precedence, and the score branch dynamically gauged the proximity of the anticipated score.

Zheng et al. [44] for violence detection combined video analysis from non-equidistantly selected frames and audio from those videos. Feature extraction was performed on RGB video and audio using a transformer encoder architecture network (to extract deep temporal-spatial features) and VGGish (to extract audio features). These extracted features were then run through an LSTM to extract temporal features. Cheng et al. [94] proposed a model called *VARN*, which consisted of pseudo-3D convolutional networks pre-trained with UCF101 and AudioNet (pre-trained CNN starting from SoundNet). The fused image and audio elements were passed to two fully connected neural networks. The reasoning network generated a vector of size 7X1, representing seven types of conflict events (no conflict, personal verbal conflict, personal physical conflict, personal physical conflict with weapons, group verbal conflict, group physical conflict, and group physical conflict with weapons). The so-called predicted network was responsible for predicting the degree of danger of conflict events.

### 3.6. Transformer-Based Approach

Akti et al. [45] used an algorithm called ViT. The *vision transformer (ViT)* is a neural network architecture that combines elements of transformer-based vision models with self-attention. This algorithm first patches the image and extracts features from each of the patches, taking into account the position of each patch in the original image. Then, that information is passed to another layer where temporal relationships between patches are extracted, and finally, a classification is performed. In addition, in this article, a dataset with images and videos obtained from the Internet was presented.

Ehsan et al. [46] approached the extraction of feature frames by detecting the people present in the video and removing all background information using YOLO. The Farneback method was used for image optical flow calculation. *STAT*, a generative adversarial network (GAN)-based algorithm that performs the unsupervised translation of temporal motion features in video sequences to spatial image frames, was used for feature extraction. In the proposed algorithm, the STAT network consisted of a generator (G) and a discriminator (D). The generator took motion variation features extracted from video sequences and translated them into image frames. The discriminator evaluated the authenticity of the generated images compared to the real images. During training, the generator attempted to generate realistic images, while the discriminator tried to distinguish between real and generated images. After training, the STAT generator (with the generator only) was used to translate normal motion features into normal images, but failed to reconstruct violent actions. Interpretation of the difference between the original and reconstructed images allowed for the classification of human behavior as normal or violent.

Kumar et al. [95] introduced a streamlined transformer model, drawing inspiration from the recent achievements of video vision transformers in action recognition. Spatial characteristics were captured from the input frames, and temporal relationships among the selected frames were improved through tubelet embedding. These enhanced frames were then processed using various transformer layers. Typically, despite the known requirement of extensive training with large datasets for transformer models, it has been demonstrated that effective training on relatively compact datasets can be achieved by employing efficient preprocessing techniques. In addition, the authors created a new violence detection dataframe.

### 3.7. LSTM-Based Approach

Ullah et al. [47] focused on the detection of violence in industrial environments. An algorithm was proposed which used object detection for fragmentation. In addition, two types of neural networks, an LSTM and a GRU, were used to solve the gradient evanescence, both are recurrent neural networks (RNNs) that avoid the vanishing gradient in different ways. The authors also presented the architecture and the operation of the cloud distribution of their violence detection system.

## 4. Description of the Analysis of the Selected Items

Section 4 describes the techniques proposed in the selected articles, which are used in each part of the violence detection process. Current violence detection challenges are described and related work is categorized. Section 4.1 describes the different approaches of the selected articles to categorizing violence detection algorithms. Section 4.2 outlines the challenges associated with violence detection in video, following the assertions made in the selected articles. Section 4.3 expounds the content of all the datasets used in the selected articles. Section 4.4 describes each relevant frame extraction method presented in the selected articles. Section 4.5 explains each input for violence detection algorithms, as used in the selected articles. Section 4.6 gathers the different types of violence detection algorithms applied in the selected articles, as well as their combinations. In addition, graphs are used to represent the performance of the proposals (in terms of accuracy) in which the most widely used datasets have been employed. Section 4.7 reviews the articles that have studied the use and relevance of classifiers in the detection of violence.

### 4.1. Physical-Aggression-Related Work

This section groups, in Table 1, the different ways in which the selected papers have organized the related work section. From the 63 selected articles, 55 of them categorized the algorithms into *traditional methods* and *deep learning methods*. Among *traditional methods* are those that use machine learning techniques or manual feature extraction. This traditional division can be vague and imprecise given the wide variety of existing solutions. Only the eight papers cited in Table 1 categorized methods differently. There are four categories for the different approaches. The categorization proposal of this work is presented in Section 3, providing a precise breakdown of the nature of the violence detection algorithms that have been used in the literature.

### 4.2. Physical Aggression Challenges

**Figure 2:**
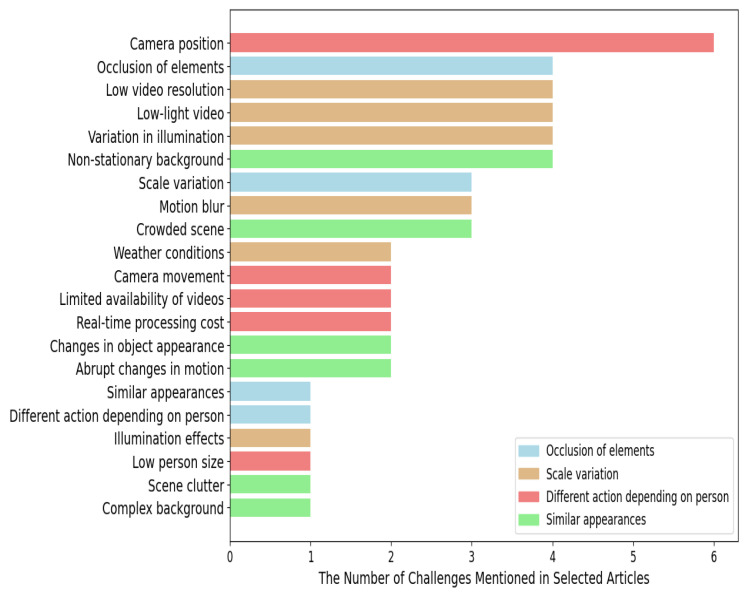
Classification and count of the types of challenges in violence detection on video.

This section explains the challenges in violence detection mentioned in the selected articles graphed in Figure 2.

The challenges have been grouped into four groups according to their nature. The challenges with red bars correspond to those related to *hardware and real-time considerations*, those with blue bars to challenges related to *problems of detection and monitoring*, those with yellow bars to those related to *image quality and lighting conditions*, and those with green bars to those related to *changes in the stage and environment*. Each of the challenges is now explained by subgroup.

Firstly, let us address *problems with detection and monitoring*. *Occlusion of elements* refers to the challenge of identifying physical aggression in videos when the objects or persons involved are partially or completely hidden by other elements in the scene. *Scale variation* is the challenge of detecting physical aggression in videos when the objects or people involved may vary in size in the scene. This means that aggression events can occur at different distances from the camera, making accurate detection difficult. *Different action depending on person* refers to the challenge of identifying physical assault in videos when actions vary depending on the person involved. *Similar appearances* refers to the similarity between violent and non-violent actions such as hugging or playing.

Secondly, let us address the challenge associated with *image quality and lighting conditions*. *Variation in illumination* refers to fluctuations in lighting conditions, such as shadows or light changes, which make it difficult to accurately detect physical assault in video surveillance systems, as they can affect the visibility and quality of captured images. *Low-light video* refers to capturing physical assault in environments with poor lighting, such as nighttime spaces or poorly lit areas. *Low video resolution* refers to the detection of physical assault in low-resolution video, which poses difficulties, as details are sparse and image quality is limited, leading to lower accuracy in identifying violent events. *Motion blur* refers to the blurring caused by rapid motion in video images. This hinders the detection of physical assault, as it can distort the key features of violent events. *Weather conditions* refers to adverse weather conditions, such as heavy rain or fog, which can hinder the detection of physical assault in video surveillance systems by reducing visibility. *Illumination effects* refers to variations in illumination, such as reflections, shadows and drastic light changes, which can distort the appearance of violent events in video images.

Thirdly, let us describe the challenge associated with *hardware and real-time considerations*. *Camera position* refers to the difficulty of training a model using images captured from various perspectives, as the algorithm may not generalize properly and may face difficulties in recognizing violent events in conditions that differ from the training perspectives. *Real-time processing cost* refers to the detection of physical assaults in real time, which can be costly in terms of computational resources. This may limit the effective implementation of real-time security systems. *Limited availability of videos* refers to the fact that obtaining sufficiently large and varied datasets of physical assaults can be challenging, as these events are relatively rare and may not be widely documented on video. *Camera movement* refers to camera movement during recording, which can make the accurate detection of physical assault difficult, as changes in perspective during the assault can affect the appearance of violent events. *Low person size* refers to the varying location of the person with respect to the camera, which may result in a relatively small physical aggression size in the video, posing a challenge to accurate detection.

Finally, the challenges associated with *changes in the scene and environment*. *Non-stationary background* refers to the presence of a non-stationary background in the video, such as people or objects constantly moving behind the action. *Crowded scene* refers to the presence of a crowd in the scene, which can make it difficult to focus on aggression at a specific point in the footage, posing a challenge to accurate detection. *Abrupt changes in motion* refer to sudden and abrupt changes in the movement of the persons involved in the physical aggression. *Changes in object appearance* refer to the modification of appearance of objects or items involved in the aggression. *Complex background* refers to the existence of textures, patterns, or complex elements in an image that may hinder detection of aggression. Finally, *scene clutter* refers to visual clutter in a scene due to a large number of objects, elements, or details.

In summary, the described challenges, which have been categorized into four sub-groups, encompass the current challenges encountered in the identification of violence on video.

### 4.3. Physical Aggression Datasets

Detecting violence in video presents a challenge because of the infrequency of physical aggression compared to everyday activities, such as observing individuals engaged in sports or operating a vehicle, despite its prevalence in all societies. Consequently, recording such incidents is not a regular affair, although the proliferation of security cameras over time has contributed to the growing availability of recordings. Therefore, the creation of datasets that gather instances of physical assault, as well as non-violent events that may resemble incidents of assault, are of great importance for effective model training. This section describes the physical aggression datasets that have been used in the selected articles, sorted in descending order by the number of articles that have employed them.

The Hockey Fights dataset [96] has been the most used dataset among the selected articles. It contains action videos from field hockey games of the National Hockey League (NHL). Action Movies dataset [96] is the second most used dataset and contains action movie scenes. Violent Flow [97], also known as violent crowd, contains YouTube videos of real-world crowds with audio. Real World Fight-2000 (RWF-2000) [17] contains surveillance cameras fights in real-world scenes. Real Life Violence Situations (RLVS) [98] contains real-life violence situations obtained from YouTube videos. UCF-Crime Selected (UCFS) [99] contains long untrimmed surveillance anomaly videos with audio which cover 13 categories, including: abuse, arrest, arson, assault, accident, burglary, explosion, fighting, robbery, road accident, shooting, stealing, shoplifting, and vandalism.

The BEHAVE dataset [100] contains long video camera recordings with bounding boxes in violent and non-violent events. The surveillance camera dataset [101] contains real day and night scenes from a surveillance camera, with indoor and outdoor footage. The XD-Violence Selected (XD-V) dataset [93] contains six violent classes (abuse, car accident, explosion, fighting, riot, and shooting) from movies, sports, games, hand-held cameras, CCTV, car cameras, etc. It contains audio and long videos. The AIRTLab dataset [82] contains 60 non-violent clips from two different points of view and 120 violent clips from two different points of view. All the clips were recorded in the same room, with natural lighting conditions, placing two cameras into two different spots. The industrial surveillance dataset [47] contains varied scenes from Google and YouTube, recorded in different stores, offices, and petrol pumps linked to industries. Most of the actions are away from the center point from the camera and the fps varies as in other surveillance datasets.

The human violence dataset [65] contains violent actions from movie promotion videos on YouTube. There are multiple categories for each video, such as combat, physical contact, weapon possession, and blood. The target dataset [61] contains drone surveillance videos of diversified views and the unconstrained environment from different heights. The dataset developed in [89] contains the authors’ own recorded videos with two types of single-person violent human actions (punching, kicking) carried out two times by 20 different people in varying plots: different backgrounds and lighting conditions, outdoors and indoors, from different angles, outdoors and indoors with different clothes. Srivastava et al. [91] developed a dataset which contains drone images recorded from 2 to 18 m by actors of both genders, between 17 and 30 years old and of different heights. The HD (high definition) [91] dataset consists of high-definition (HD) video segments lasting 2 h each, with a resolution of 1280 × 720 pixels. The dataset includes specific timestamps for violence occurrences within these videos, while the rest of the content portrays normal conversations and non-violent scenes.

The conflict event dataset [94] contains videos divided into 51 action categories. The dataset developed by Adithya et al. [72] contains diverse violence videos recorded with a drone. The dataset developed by Kumar et al. [95] contains RLVS dataset videos, clips collected from YouTube, and relevant clips from GitHub. In the non-violence part, it contains videos of waving, walking, dancing, jumping, exercising, performing house chores, and other such actions that are common in an indoor setting. VSD2015 [102] is an extension of the LIRIS-ACCEDE dataset composed from action movies. The AVA-Kinetics dataset [103] contains 80 different human actions. If only violent and non-violent actions are considered, there is a total of 13 actions. Media Eval VSD-2014 [104] contains seven action movie scenes, including fights, blood, fire, guns, cold weapons, car chases, gory scenes, gunshots, explosions, and screams.

Mahalle et al. developed a dataset [43] which contains collected YouTube videos converted to audio. This dataset also includes other types of aggression, apart from physical assault. It contains 31 categories, including gunshots, screaming, explosions, and arguments. The Social Media Fight Images (SMFI) [45] dataset contains images and video frames collected from Twitter, Google, and NTU CCTV-Fights. The dataset developed by Naryanov et al. [90] contains real aggressive behavior and bullying on the internet and on social network videos. The dataset developed by Rachna et al. [105] contains YouTube videos, stock films, and self-recorded violent videos. The Violent Clip Dataset (VCD) [83] contains 37 Hollywood movies and 30 YouTube video clips. The dataset created by Hung et al. [42] includes volunteers acting out different scenarios, such as elderly patients with lower limb disabilities.

A summary of the characteristics of the selected datasets is represented in Table 2 [32]. In conclusion, a diverse variety of datasets from diverse sources and with different size and content are publicly available. This diversity is highly advantageous in the study of physical aggression detection with specific requirements.

### 4.4. Methods of Selection of Relevant Frames

Relevant frame extraction consists in the selection of frames that may contain violence, so that the algorithm does not have to process all recorded frames. This significantly reduces the computational and economic cost [64,87]. This section explains the relevant frame extraction methods used in the selected articles. Three categories have been created: image variation, systematic sampling, and object detection.

First, the methods for selecting characteristic frames based on *image variation* are presented. Mahmoodi et al. [63] used an image segmentation method called SSMI which only selects frames when there is variation in brightness, contrast, or structure. Magdy et al. [37] used the Farneback technique, which is an optical flow method that estimates motion in a sequence of images by comparing local texture and brightness patterns, generating a map of motion vectors and extracting only those where there is variation. Bi et al. [73] used a characteristic frame selection technique based on optical flow, reducing the number of frames processed by 1/k; in addition, based on the number of frames selected, the authors filtered out non-violent actions such as hugs. Chen et al. [38] used a method to split long videos into smaller videos through the difference in brightness between adjacent frames, which could indicate a change in the action of the video; where the maximum variation in brightness determines the segmentation. Ahmed et al. [64] performed the difference between two matrices of frames with threshold being a mathematical process that compares corresponding pixels in two images and marks as active those pixels whose difference exceeds a predefined threshold value. Jaiswal et al. [87] used geometric fragmentation methods, focusing on the spatio-temporal variation in the frames.

We now turn to the methods within the *systematic sampling* category of characteristic frame selection; these methods are based on selecting a certain number of frames out of a certain number of frames or per unit of time, thus continuously processing the incoming video, albeit in a lighter way than analyzing all frames all the time. Wintarti et al. [39] selected 20 frames from each video sequence at random. Ref. [57] selected 20 frames randomly from each frame packet, further converting the images to square by choosing any area of the dataframe in between (random). Asad et al. [53] took inputs from two video frames at times *t* and *t* + 1 at regular intervals. Kumar et al. [95] selected F number of frames per second at equidistant intervals. Zheng et al. [44] selected 16 frames from the videos randomly.

Finally, the methods in the *object detection* category. Several articles in this category used YOLO [46,68,70,78,105], which is a widely used object detection algorithm in images and videos that uses a convolutional neural network to identify and locate objects in real time. Rachna et al. [105] and Freire et al. [74] also did this in conjunction with DeepSort, which is a video object tracking algorithm. Hung et al. [42] used OpenPose, which is a library and a set of deep learning models used for human body keypoint detection. Singh et al. [60] used OpenCV (Open Source Computer Vision Library), which is a widely used open-source library in computer vision that provides a wide range of tools and functions to perform tasks such as object detection and object tracking. Naik et al. [89] and Ullah et al. [48] used Mask-RCNN, which is a convolutional neural network architecture used for object detection and semantic segmentation in images. Finally, Ullah et al. [47] proposed a lightweight CNN architecture for object detection and frame selection.

Thus, choosing the right keyframes is crucial for making real-time violence detection systems efficient and cost-effective, with many alternatives to choose from.

### 4.5. Violence Detection Algorithm Input

**Figure 3:**
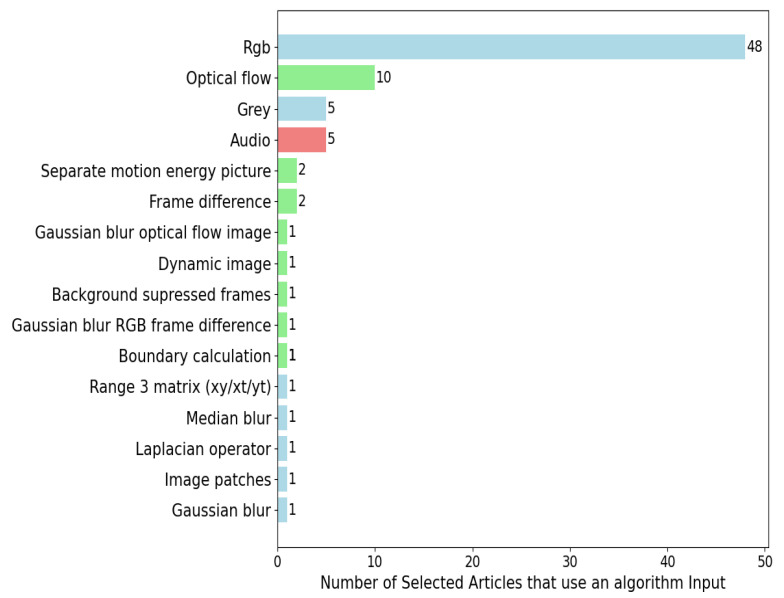
Count and categorization of the types of algorithm inputs used in the selected articles, grouped by category.

After obtaining a dataset of labeled videos for algorithm training and deciding to use a keyframe extraction method, the next step is to select what information is provided to the algorithm. This choice is crucial because it enables the algorithm to extract the relevant features and train to classify violence or non-violence. There are various methods for inputting video information to the algorithm, including RGB video, grayscale video, optical flow, and more. This section explains each input type, in descending order in accordance with the frequency of use among the selected articles. The inputs used in the video violence detection algorithms are shown in Figure 3. They have been divided into three categories: the blue bars correspond to the inputs from the video itself, the green bars correspond to the inputs from the movement, and the green bars correspond to the audio input.

- **RGB videos**: This input consists of the red, green, and blue color channels of a video. It is a standard representation of images and videos where each pixel has intensity values in these three channels.
- **Optical flow**: It represents the speed and direction of object motion in a video. It is obtained by calculating pixel displacement between consecutive frames, enabling the detection of motion in the video.
- **Video audio**: The audio from the video can be an important input for violence detection, as it may contain sound cues associated with violent events such as screams.
- **Grayscale videos**: A grayscale video contains only luminance information and lacks color data. It is useful when the analysis focuses on changes in luminance intensity rather than colors.
- **Frame difference**: This input is based on the intensity difference between successive frames. It can be useful for detecting abrupt changes or motion in a video.
- **Separate motion energy picture**: Visual representation highlighting areas of significant motion energy in a video, created by calculating and emphasizing regions with rapid pixel intensity changes across frames. It helps detect and analyze movement in videos, aiding tasks such as motion detection and event identification.
- **Background-suppressed frames**: Frames where the background has been removed or suppressed, leaving only foreground moving objects.
- **Boundary calculation**: Involves determining and highlighting edges or boundaries within a video, aiding in the identification of object contours and spatial structures. It helps detect and analyze object shapes and boundaries in video frames.
- **Dynamic image**: Focuses on the motion of salient objects in the video, combining and averaging the background pixels with the motion patterns while retaining the long-term kinetics.
- **Gaussian blur RGB video**: Input applies a Gaussian blur filter to the individual red, green, and blue color channels of a video. This smoothing technique reduces noise and fine details, resulting in a softened, less detailed version of the original video frames.
- **Gaussian blur optical flow**: Input applies a Gaussian blur filter to the optical flow field in a video. This helps reduce noise and enhances the smoothness of the optical flow vectors, making it more suitable for motion analysis and detection tasks by reducing spurious motion artifacts.
- **Gaussian blur RGB difference between images**: Involves computing the difference in color information between two images in an RGB video, and then, applying a Gaussian blur to this difference. This technique is used to reduce noise and emphasize substantial changes in color or objects between consecutive frames, aiding in the detection of significant visual alterations in videos.
- **Image patches**: Divides an image or video frame into smaller, rectangular regions known as patches. Each patch represents a localized portion of the image.
- **Laplacian operator**: Mathematical filter applied to an image to enhance its edges and fine details. It calculates the second spatial derivative of the image, emphasizing regions where pixel values change rapidly. This operator is commonly used in image processing for edge detection and feature extraction tasks, highlighting variations in intensity.
- **Median blur**: Image processing technique that replaces each pixel’s value with the median value within a defined neighborhood or window. It effectively reduces noise and removes small-scale details in an image, resulting in smoother regions while preserving edges and larger structures. Median blur is commonly used for denoising and enhancing image quality.
- **Range 3 matrix (xy/xt/yt)**: Typically represents spatiotemporal information in a video. It is a 3D matrix that encompasses three dimensions: spatial (x, y), temporal (t), and frequency domain (temporal frequency or optical flow). This representation is used for advanced video analysis and often involves capturing intricate details of motion and change over time, making it valuable for tasks like action recognition and tracking.

All things considered, the variety of input types that can be introduced to the algorithm is very broad, each with a different approach to the feature extraction that the algorithm is to perform.

### 4.6. Violence Detection Algorithms

This section presents the algorithms used in the selected articles to detect violence. The process of detecting violence is divided into two phases: *phase 1* and *phase 2*. There are algorithms that do not combine with other algorithms, so they only have *phase 1*. Section 4.6 displays the number of algorithms from each category used in phase 1 and phase 2 in the selected articles for violence detection. It also shows the number of articles that use a certain combination of algorithms in both phase 1 and phase 2 together. Section 4.6.2 presents the accuracies obtained using three of the five most used datasets in violence detection.

#### 4.6.1. Analysis of the Types of Algorithms Used in the Selected Articles

**Figure 4:**
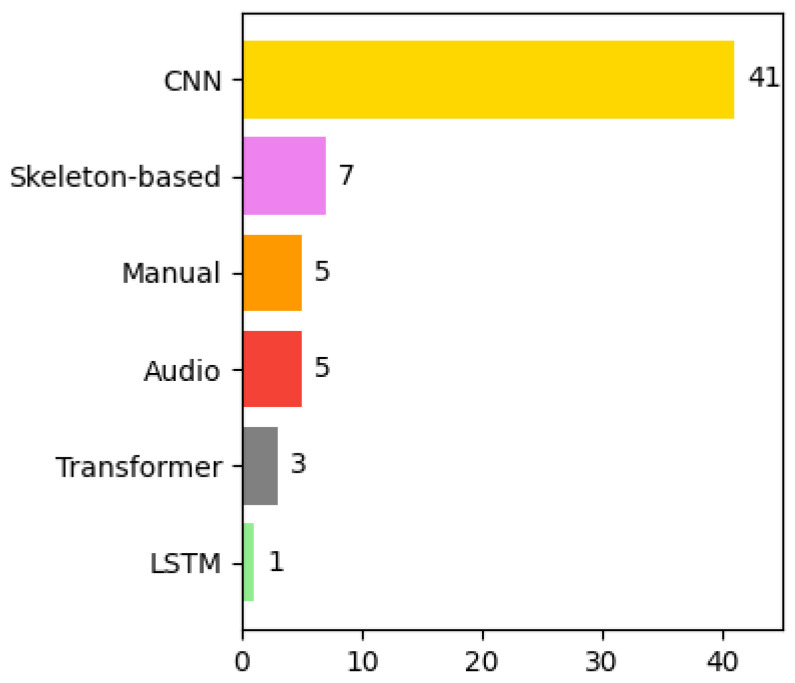
Count of the types of algorithms used in violence detection phase 1 in the selected articles grouped by category.

**Figure 5:**
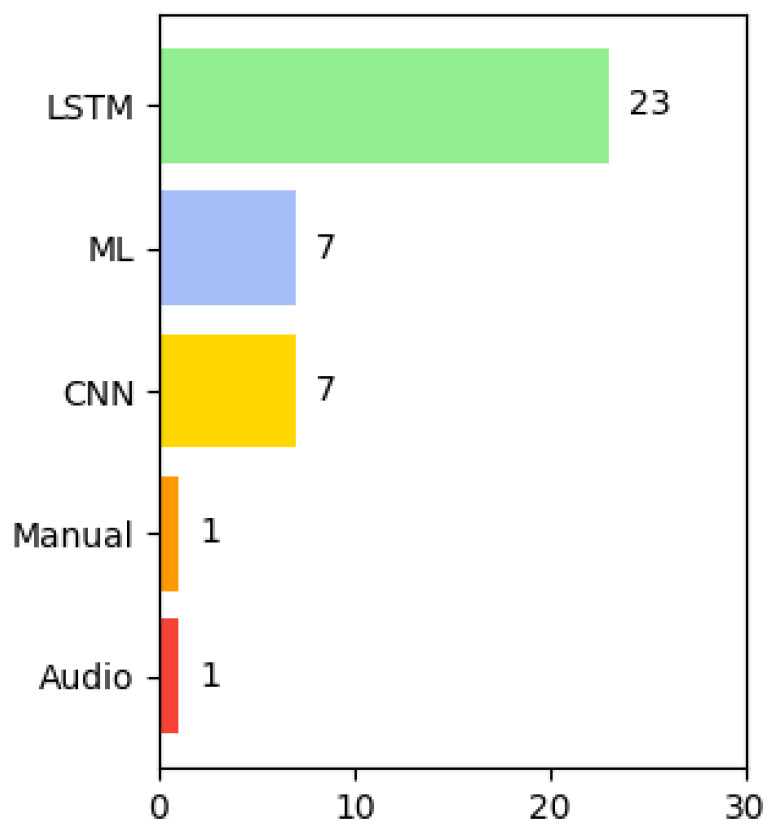
Count of the types of algorithms used in violence detection phase 2 in the selected articles grouped by category.

**Figure 6:**
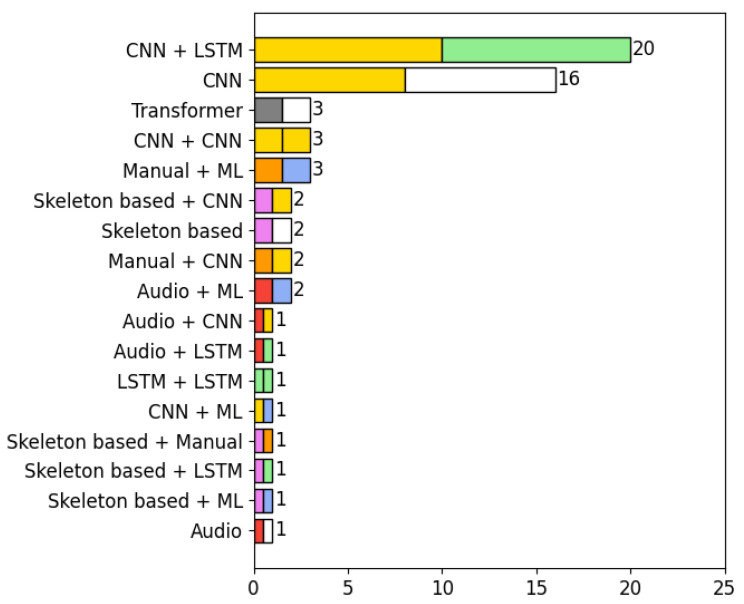
Count of the types of algorithm combinations used in the selected articles grouped by subcategory. Colors: Yellow corresponds to the use of CNNs, purple to skeleton-based algorithms, orange to manual techniques, red to those utilizing audio, gray to those employing transformers, and green to LSTM.

**Figure 7:**
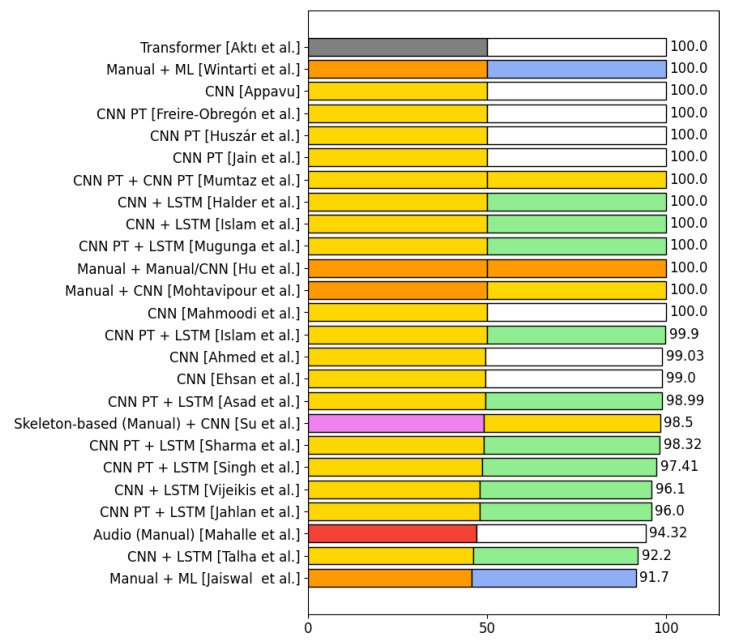
Accuracy obtained by selected items in the Action Movies dataset. Colors: Yellow corresponds to the use of CNNs, purple to skeleton-based algorithms, orange to manual techniques, red to those utilizing audio, gray to those employing transformers, and green to LSTM. Citations in descending order of accuracy: [29,35,39,43,45,49,50,53,56,57,58,59,60,62,63,64,66,71,74,76,77,86,87,88,92].

**Figure 8:**
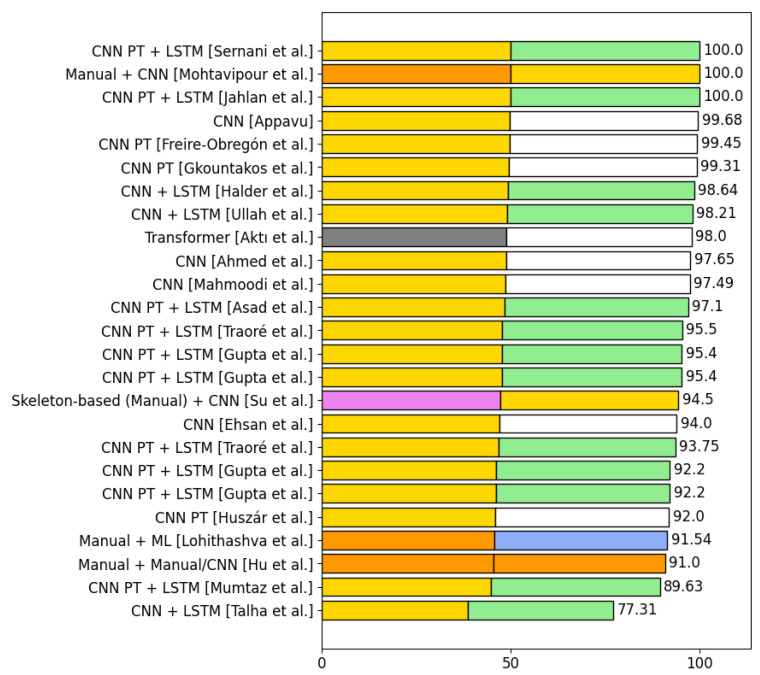
Accuracy obtained by selected items in the Violent Flow dataset. Colors: Yellow corresponds to the use of CNNs, purple to skeleton-based algorithms, orange to manual techniques, red to those utilizing audio, gray to those employing transformers, and green to LSTM. Citations in descending order of accuracy: [35,40,45,47,50,51,53,55,57,58,63,64,66,71,74,75,76,82,86,88,92].

**Figure 9:**
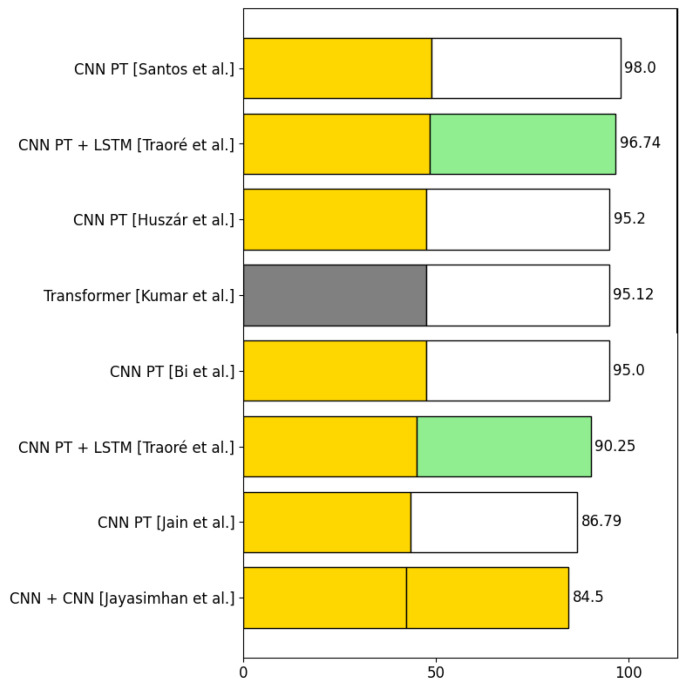
Accuracy obtained by selected items in the Real Life Violent Scenes Dataset. Colors: Yellow corresponds to the use of CNNs, purple to skeleton-based algorithms, orange to manual techniques, red to those utilizing audio, gray to those employing transformers, and green to LSTM. Citations in descending order of accuracy: [51,67,73,76,77,81,95].

In this section, the most commonly used algorithms in *phase 1* and *phase 2* are presented. It is also discussed which combinations of algorithms are most commonly used. Algorithms are grouped by category, depending on the algorithm type, CNN, LSTM, manual feature, skeleton-based, transformer, and audio-based, without differentiating between pre-trained algorithms or not, in the case of CNN, and without differentiating whether the algorithm used is based on deep learning or manual techniques in the case of skeleton-based algorithms and audio-based algorithms. Figure 4, Figure 5, Figure 6, Figure 7, Figure 8 and Figure 9 use a consistent color code to identify the type of algorithm used by the selected studies. Yellow corresponds to the use of CNNs, purple to skeleton-based algorithms, orange to manual techniques, red to those utilizing audio, gray to those employing transformers, and green to LSTM. Figure 4 represents a count of the algorithms used in *phase 1* in the selected articles, grouped by category. In this case, it can be observed that the clear majority use CNN in comparison to other types of algorithms. In addition, skeleton-based, manual, and audio algorithms have a similar number of citations. Since LSTMs extract temporal features, their use in the first phase is not very extensive, unlike in the second phase, as seen below.

Figure 5 represents a count of the algorithms used in *phase 2* in the selected articles, grouped by category. In this case, it can be observed how LSTM is the main algorithm used in *phase 2* (with the intention of extracting temporal features). Traditional machine learning techniques and CNN are the second most used, more than three times less than LSTM.

Figure 6 shows the most used model architecture combinations in the selected articles, grouped by category. If the work just uses *phase 1*, the second phase is represented with no color. The most used combination is CNN + LSTM, followed by the exclusive use of CNN. To a much lesser extent, other combinations combining traditional machine learning algorithms, skeleton-based, audio-based, etc., have been used.

Thus, it has been possible to observe the most used algorithms in phases 1 and 2 of the process of detecting violence in videos, as well as the most used combinations.

#### 4.6.2. Accuracies Obtained with Violence Detection Algorithms

In this section, the accuracies obtained by the selected works for three of the five most widely used datasets in the literature are presented: Action Movies dataset, Violent Flow dataset, and Real Life Videos dataset. These were described in Section 4.3.

Figure 7 shows how a large number of the selected papers have very high accuracies. Only 3 papers out of 25 in total have accuracies below 95%. While it is true that the algorithms used are getting better and better as the state of the art advances, the dataset itself has something to do with it. This is because the Action Movies dataset contains scenes taken from action movies, where the focus of the scene, its lighting, the image quality, and the shot make it much easier to detect violence. This is to emphasize that while the use of widely used datasets makes it much easier to compare results from different works, by obtaining results that tend to have such a high detection rate, it is difficult to draw conclusions about the true quality of the algorithm.

Figure 8 and Figure 9 refer to the accuracies obtained in the selected works in the datasets Violent Flow dataset and Real Life Violent Scenes dataset, respectively. It can be observed that the accuracies obtained are already more distributed towards lower values, since these datasets contain real scenes, which are more difficult to detect. Even so, multiple works obtain excellent results. The use of only CNN and the combination of CNN + LSTM is, as mentioned above, the most widespread, although it does not always obtain such good results. Transformers have been little used in the recent state of the art, although they obtain good results. It is highlighted that although these are some of the most used datasets in the state of the art, many articles train the algorithms with their own datasets, which makes a comparison between different algorithms difficult.

### 4.7. Classifiers in Violence Detection

After phases 1 and 2 of violence detection, the process ends with a classifier that determines whether the video is violent or not; this is, therefore, a very important process. Although the improvement in the detection of violence in video has mainly focused on the optimization of the algorithms that perform the extraction and training of the features, there are articles that have tried to verify how the results of the algorithms vary when using different classifiers, considering the classification a decisive moment in the process.

Freire-Obregón et al. [74] tested the effectiveness of several machine learning classifiers, concluding that the use of videos from which the context of the scene was removed suffered a loss in accuracy of between 2 and 5%; moreover, this percentage is independent of the degree of context removed. It was highlighted that LSVM and LR are effective classifiers for violence detection in videos, outperforming tree-based approaches, where XGBoost is the most effective tree-based approach, especially on the AVD dataset due to its sequential tree-building approach. Jahlan et al. [57] used three different classifiers to test their effectiveness. Islam et al. [62] tested different variations of the last layers prior to the densely connected layers to observe the variation in the results obtained in violence classification. Hu et al. [88] proposed, in addition to the use of an SVM as a classifier, to use a lightweight CNN. All in all, the video violence classification process is based on the generation of a result, usually binary, where the event is classified as violent or non-violent. There are different types of classifiers that can be mainly divided between techniques based on deep learning or machine learning. Finally, there are works that have given importance to the classifier selection by checking how the results of violence detection vary.

## 5. Conclusions and Future Work

Physical aggression is a serious and widespread concern in our society, affecting individuals globally and impacting nearly every aspect of life. This influence extends not just to the immediate victims but also to their families, the broader community, and the day-to-day development of the nation, even having consequences on the economic environment [1]. Potential solutions have been studied, although real-time violence detection is the quickest and most immediate safeguard for victims [14].

This paper, which is a continuation of a systematic mapping study, consists of a review based on 63 selected articles dealing with violence detection in videos using artificial intelligence. The basic steps involved in violence detection have been described, and the 63 selected articles have been briefly outlined, grouped according to the type of violence detection algorithm used.

An extensive analysis has been conducted, describing the 28 datasets used in the selected articles, with Hockey Fights being the most utilized. Twenty-one challenges, mentioned in the selected articles, are yet to be resolved in violence detection, with the camera position being the most frequently described challenge. Twenty-one methods for keyframe extraction have been compiled and categorized into three different categories. Furthermore, the types of algorithms and the most commonly used combinations in the selected articles have been analyzed, where the combination of convolutional neural networks along with the use of long short-term memory is the most used, with good results highlighted for this combination. Lastly, articles investigating the improvement in violence detection according to the classifier used are analyzed.

As future work, it is proposed to develop algorithms based on the combination of CNN and LSTM, given that they obtained excellent results in the analyzed articles. It is also intended to apply trustworthy artificial intelligence techniques to make the decision-making process of these algorithms more comprehensible, in line with the requirements for the use of AI, as established by the European Union in recent years [106].

## Figures and Tables

**Table 1:** Related work categorizations from selected papers.

Work Reference	Categorization Criteria	Categories
[76]	Algorithm’s approach	Modeling normal patterns, multiple instance learning, supervised learning
[73]	Algorithm’s approach	Semantic segmentation algorithms, keyframe extraction methods, violence detection models
[64]	Algorithm’s approach	Trajectory-based methods, non-object-centric-based methods, deep-learning-based methods
[83]	Algorithm’s nature	Violent video recognition approach, self-supervised learning, knowledge distillation
[60]	Algorithm’s nature	Convolutional neural networks, motion-detection-based, other machine learning algorithms
[41]	Stage of the detection process	Human pose estimation, action recognition
[69,86]	Spatial focus	Local approach, global approach, deep learning methods

**Table 2:** Summary of dataset characteristics in selected articles [32].

Name	Original Citation	Year	Number of Clips	Mean Frames or Mean Duration of Clips	Frame Rate (FPS)	Video Quality of Clips	Number of Citations
Hockey Fights	[96]	2011	1000	50 frames	20–30	720 × 576	38
Action Movies	[96]	2011	200	49 frames	20–30	515 × 720	27
Violent Flow/Violent Crowd	[97]	2012	246	N.S.	25	320 × 240	24
Real World Fight-2000 (RWF-2000)	[17]	2021	2000	5 s	30	Multiple	13
Real Life Violence Situations (RLVS)	[98]	2019	2000	5 s	20–30	480–720	8
UCF-Crime Selected (UCFS)	[99]	2018	1900	7247 frames	N.S.	N.S.	5
BEHAVE	[100]	2010	4	76,800 frames	25	640 × 480	4
Surveillance camera	[101]	2019	300	2 s	Multiple	Multiple	3
XD-Violence Selected (XD-V)	[93]	2020	4754	N.S.	N.S.	N.S.	3
AIRTLab	[82]	2020	350	5.63 s	30	1920 ×1080	3
Industrial Surveillance	[47]	2021	300	5 s	20–30	Multiple	2
Human Violence	[65]	2021	1930	30–100 s	30	1280 × 720	1
Target	[61]	2022	150	5–10 s	60	1080p	1
Own [89]	[89]	2021	273	N.S.	10	N.S.	1
Own [91]	[91]	2021	N.S.	610 s	60	1080p	1
HD (high definition)	[91]	2022	2	2 h	N.S.	1280 × 720	1
Conflict event	[94]	2021	6849	N.S.	N.S.	N.S.	1
Own [72]	[72]	2023	20	N.S.	N.S.	N.S.	1
Own [95]	[95]	2023	1112	N.S.	N.S.	N.S.	1
VSD2015	[102]	2015	10,900	8–12 s	N.S.	N.S.	1
AVA-Kinectics dataset	[103]	2020	430/143	15 min	N.S.	N.S.	1
Media Eval VSD-2014	[104]	2014	N.S.	N.S.	25	N.S.	1
Own [43]	[43]	2021	60	30 s	Audio	Audio	1
Social Media Fight Images (SMFI)	[45]	2022	5691	N.S.	N.S.	N.S.	1
Own [90]	[90]	2021	2093	34.41 s	N.S.	N.S.	1
Own [105]	[105]	2022	106	N.S.	N.S.	N.S.	1
Violent Clip Dataset (VCD)	[83]	2022	7279	8–12 s	N.S.	N.S.	1
Own [42]	[42]	2021	1500	3–6 s	13–15 s	N.S.	1
